# Genomic insights into the spread of vancomycin- and tigecycline-resistant *Enterococcus faecium* ST117

**DOI:** 10.1186/s12941-025-00806-7

**Published:** 2025-06-11

**Authors:** Marie Brajerova, Otakar Nyc, Pavel Drevinek, Marcela Krutova

**Affiliations:** Department of Medical Microbiology, Second Faculty of Medicine, Charles University and Motol University Hospital, Prague, Czech Republic

**Keywords:** Whole-genome sequencing, antimicrobial resistance, Long-read sequencing, Plasmids, Structural variants, Daptomycin

## Abstract

**Background:**

Since the incidence of vancomycin-resistant enterococci (VRE) is increasing and treatment options remain limited, we aimed to investigate the epidemiology of vancomycin- and tigecycline-resistant enterococci in a university hospital using whole genome sequencing (WGS).

**Methods:**

Between April and December 2021, 102 VRE isolates were collected from a single tertiary care hospital in the Czech Republic. Forty selected isolates underwent antimicrobial susceptibility testing and WGS (Illumina short reads and long reads with MinION in selected isolates).

**Results:**

All *Enterococcus faecium* isolates were resistant to ampicillin, carrying the PBP5_Met485Ala, PBP5_Glu629Val, and fluoroquinolones carrying the GyrA_Ser83Ile and ParC_Ser80Ile substitutions. The *vanA* operon was found on pELF2-like plasmids and plasmids carrying *rep*17 and/or *rep*18b genes. The novel Tn*1546* structural variants were identified in *vanA*-carrying isolates. The *vanB* operon was located on the chromosome within a Tn*1549* structural variant. Linezolid resistance was detected in one isolate carrying the 23S rDNA_G2576T substitution. Twenty-two isolates were resistant to tigecycline (*tet*(L), *tet*(M) and *rpsJ*_del 155–166 or RpsJ_Lys57Arg). Discrepancies between phenotypic and genotypic resistance profiles were observed for daptomycin (RpoB_Ser491Phe), trimethoprim/sulfamethoxazole (*dfrG* gene), nitrofurantoin (NmrA_Gln48Lys substitution without the EF0404 and EF0648 genes) and tetracycline (truncated TetM). The two multilocus sequence typing (MLST) schemes identified different numbers of STs: 5 STs, with ST117 as the predominant one (*n* = 32, 80%), versus 10 STs, with ST138 (27.5%), ST136 (25%), and ST1067 (20%) being the most frequent, respectively. The whole genome MLST revealed clonal clustering (0–7 allele differences) among isolates of the same ST. When comparing ST117 isolates from our study with 2,204 ST117 isolates from 15 countries, only one Czech isolate clustered closely with strains from Germany and the Netherlands, differing by just 16 alleles.

**Conclusions:**

The spread of *E. faecium* isolates ST117 resistant to vancomycin and tigecycline was identified. The discrepancies between resistance genotypes and phenotypes highlight the importance of combining molecular and phenotypic surveillance in antimicrobial resistance monitoring.

**Supplementary Information:**

The online version contains supplementary material available at 10.1186/s12941-025-00806-7.

## Background

*Enterococcus faecium* is an opportunistic Gram-positive bacterium capable of causing severe infections, including endocarditis, bacteraemia and urinary tract infections, particularly in high-risk groups such as immunocompromised or critically ill patients. Recognized as one of the ESKAPE pathogens, vancomycin-resistant enterococci (VRE) are categorized among the “High group” on the World Health Organization Bacterial Priority Pathogen List [1]. Notably, the latest European Antimicrobial Resistance Surveillance Network (EARS-Net) report highlights an increasing incidence of invasive vancomycin-resistant enterococci (VRE) infections from 2018 to 2022, underscoring their growing clinical significance [2].

The vancomycin resistance is associated with the presence of the *van* operon in enterococci and was first identified in the mid-1980s [3]. To date, nine distinct variants of the *van* operon have been detected, the *vanA* and *vanB* operons being the most prevalent in clinical settings [4]. These operons are carried on mobile genetic elements, enabling horizontal transfer [5]. The *vanB* operon is primarily carried on the transposon Tn*1549*, located on the chromosome [6], whereas the *vanA* operon is typically carried on the Tn*1546* transposon on plasmids, such as Inc18, pRUM and pLG1 [7, 8]. Notably, recent reports have identified *vanA* operon on pELF-like linear plasmids using long-read whole genome sequencing (WGS) [9].

The primary risk associated with VRE lies in its resistance to multiple antibiotics, significantly limiting antibiotic treatment options [5]. Currently, VRE infections are treated with last-resort antibiotics such as linezolid, tigecycline and daptomycin [5]. Data on linezolid, tigecycline and daptomycin resistance remain limited to individual studies; susceptibility testing results for these antibiotics are not included in the EARS-Net surveillance. The most common mechanism of linezolid resistance in enterococci is the G2576U mutation in the 23S ribosomal RNA [10]. Additionally, linezolid resistance can be encoded by *optrA*, *poxtA* and *cfr* genes, which can be rapidly spread *via* mobile genetic elements, especially under selective conditions such as the use of antibiotics in the hospital environment [11].

Tigecycline resistance has been linked to various mechanisms, including mutations in the *rpsJ* gene or overexpression of both *tet*(L) and *tet*(M) genes [12, 13]. Similarly, multiple mechanisms for daptomycin resistance have been described, such as mutations in the LiaFSR system, a cardiolipin synthase *cls* and in *yycFG* [14]. Recently, mutations in the *rpoB* gene have been described as a novel mechanism of daptomycin resistance [15]. However, it is noteworthy that not all isolates with these mutations exhibit phenotypic resistance to daptomycin [14, 15].

In response to the increasing prevalence of VRE, we aim to characterise in detail VRE isolates at a university hospital in the Czech Republic, with a particular focus on those resistant to tigecycline and/or linezolid and/or daptomycin.

## Materials and methods

### Study design

Between April and December 2021, non-duplicated (single-patient) vancomycin resistant *E. faecium* (VREfm) isolates were collected from the patients hospitalised at Motol University Hospital, Prague, Czech Republic. The isolates were cultured from blood samples, various sites of infection or screening rectal swabs. Bacterial species identification was performed by MALDI-TOF mass spectrometry (Bruker Daltonik, GmbH, Germany). Vancomycin resistance was determined using the disk diffusion method and confirmed by E-test (bioMérieux, France). The following anonymised patient data were collected: age, sex, clinical setting, sample type and patient diagnosis.

### Antimicrobial susceptibility testing

Antimicrobial susceptibility testing for ampicillin, teicoplanin, tigecycline, linezolid and norfloxacin was performed in all VREfm isolates (*n* = 102) using the disk diffusion method (DDM, Oxoid, United Kingdom) following the European Committee on Antimicrobial Susceptibility Testing (EUCAST) clinical breakpoints [16]. Isolates showing tigecycline resistance by DDM were further retested using the broth microdilution method with freshly prepared cation-adjusted Mueller-Hinton broth (Oxoid, United Kingdom; Erba Mannheim, Germany).

Additionally, 40 VREfm isolates were selected to represent different hospital wards (*n* = 17) and/or were resistant to tigecycline or linezolid (*n* = 23). These isolates were tested using the broth microdilution for susceptibility to penicillin, ampicillin, erythromycin, clindamycin, linezolid, chloramphenicol, tetracycline, tigecycline, trimethoprim/sulfamethoxazole, gentamicin, vancomycin, teicoplanin, nitrofurantoin (Erba Mannheim, Germany), and daptomycin (with calcium ions adjusted to 50 µg/mL, Merck Group, Germany; Viamar International, Czech Republic) following the EUCAST or Clinical and Laboratory Standards Institute (CLSI) breakpoints where available (Supplementary Table 1). These 40 isolates were further characterised by WGS.

### Short read whole genome sequencing and bioinformatics

DNA was extracted using the MasterPure Gram Positive DNA Purification Kit (Biosearch Technologies, United Kingdom) with a Ready-Lyse incubation time of 60 min. DNA libraries were prepared with the NexteraXT Library Prep Kit (Illumina, USA) and sequenced on the Illumina NovaSeq6000 (Illumina, USA).

Resistance mechanisms were detected using ResFinder 4.1 [17] and LRE-finder 1.0 (Center for Genomic Epidemiology, CGE) [18]. Mutations associated with tigecycline resistance in the *rpsJ* gene [13, 19] and mutations linked to daptomycin resistance in the *rpoB*, *cls*,* yycFG* and *liaFSR* genes [15] were analysed through alignment to the *Enterococcus faecium* Aus0004 genome (NCBI accession SAMN02604218) using Geneious software v2023.0.4 (Dotmatics, USA). Additionally, replication initiation (*rep*) genes associated with plasmids were detected by PlasmidFinder 2.1 (CGE) [20].

The multilocus sequence type (MLST) was determined using two currently available schemes, the original scheme by Homan et al. [21] and the more recent scheme by Bezdicek et al. [22], both accessible at https://pubmlst.org/ [23].

The genetic relatedness of isolates was determined using whole genome multilocus sequence typing (wgMLST, 3332 loci, Bionumerics v8.1, bioMérieux, France), using 20 alleles threshold for clonal relatedness [24]. The results were visualised in a minimum spanning tree and in a phylogenetic tree using iTOL v6.9.1 [25].

To place the predominant ST117 isolates from our study into a global context, we compared 32 ST117 isolates from this study with 2,204 ST117 isolates originating from 15 countries, including the Czech Republic, downloaded from the PathogenWatch database (https://pathogen.watch/, accessed May 9, 2025; Supplementary Table 3). The ST was determined using the MLST scheme by Homan et al. [21]. The results of the wgMLST analysis were visualised in a minimum spanning tree.

### Long-read sequencing and reference genome construction

A total of 12 selected isolates were sequenced by long-read sequencing. Selection criteria included isolates carrying the *vanA* operon primarily found on plasmids, one isolate from each clonal cluster identified by wgMLST and isolates exhibiting distinct resistance profiles. DNA libraries were prepared using the Ligation sequencing gDNA kit and Native barcoding protocol (SQK-LSK109 with EXP-NBD104 and EXP-NBD114 or SQK-NBD114.24, Oxford Nanopore Technologies, UK). Sequencing was performed on R9.4.1 or R10.4.1 MinION or Flongle flow cells (Oxford Nanopore Technologies, UK).

To obtain the complete chromosome sequence, a hybrid assembly was performed using Flye v2.9.5 for long read assembly [26], Medaka v2.0.1 for polishing with long reads (available at https://github.com/nanoporetech/medaka) [27], and Polypolish v0.6.0 for polishing with short reads [28]. Plasmid sequences were assembled using Plassembler v1.6.1 [29]. Complete genomes were annotated using RAST with default settings [30]. Plasmids were compared and visualised using EasyFig v2.2.5 [31]. Tn*1546* and Tn*1549* structural variations were identified by aligning long reads to the *E. faecium* Tn*1546* sequence (GenBank accession M97297) [32] and *Enterococcus faecalis* Tn*1549* sequence (GenBank accession AF192329) [33] using Geneious software v2023.0.4 (Dotmatics, USA). Insertion sequences in Tn*1546* and Tn*1549* structural variants were detected using ISFinder [34]. The isolates with hybrid assembly are marked with an asterisk in the figures.

Raw sequence data generated in this study are publicly available in the Sequence Read Archive under BioProject PRJNA1220344.

## Results

### Patient data

A total of 102 non-duplicated VREfm isolates were collected. The median age of patients was 66.4 years, ranging between 235 days to 95 years, with 51 (50.5%) female patients.

VREfm isolates were cultured from urine (24.5%, *n* = 25), both spontaneously urinated (9.8%, *n* = 10) or samples collected *via* a permanent urinary catheter (14.7%, *n* = 15), screening rectal swabs (21.6%, *n* = 22), wound swabs (16.7%, *n* = 17), lower respiratory tract samples (13.59%, *n* = 14) and blood cultures (1.96%, *n* = 2). Additionally, one isolate was obtained from a swab taken from the hospital environment.

### Genetic relatedness of sequenced isolates

**Fig. 1:**
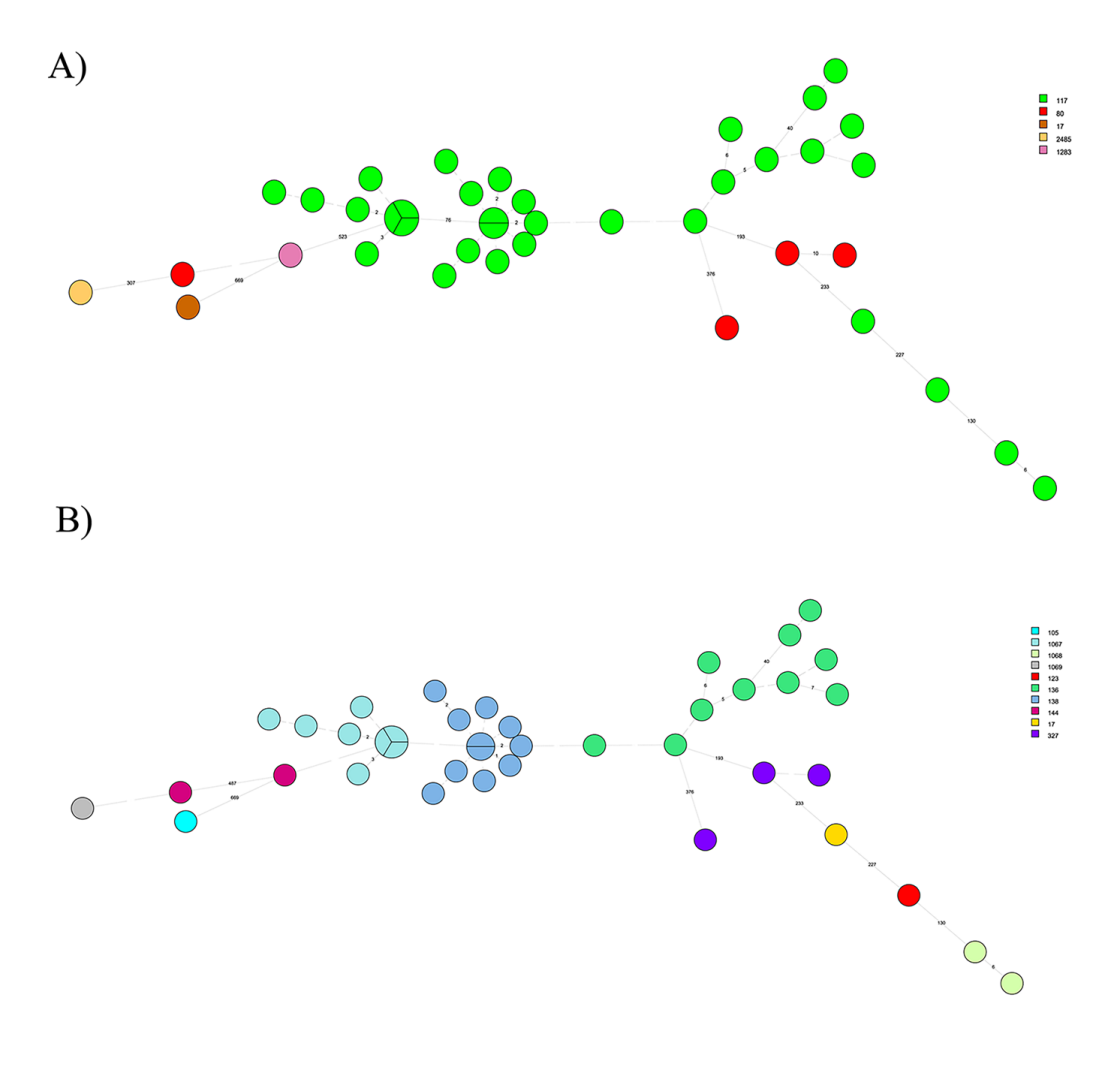
(a) A minimum spanning tree (Bionumerics v8.1, comparing 3332 loci) using the MLST scheme by Homan et al. [21]. (b) A minimum spanning tree using a newly published MLST scheme by Bezdicek et al. [22].

**Fig. 2:**
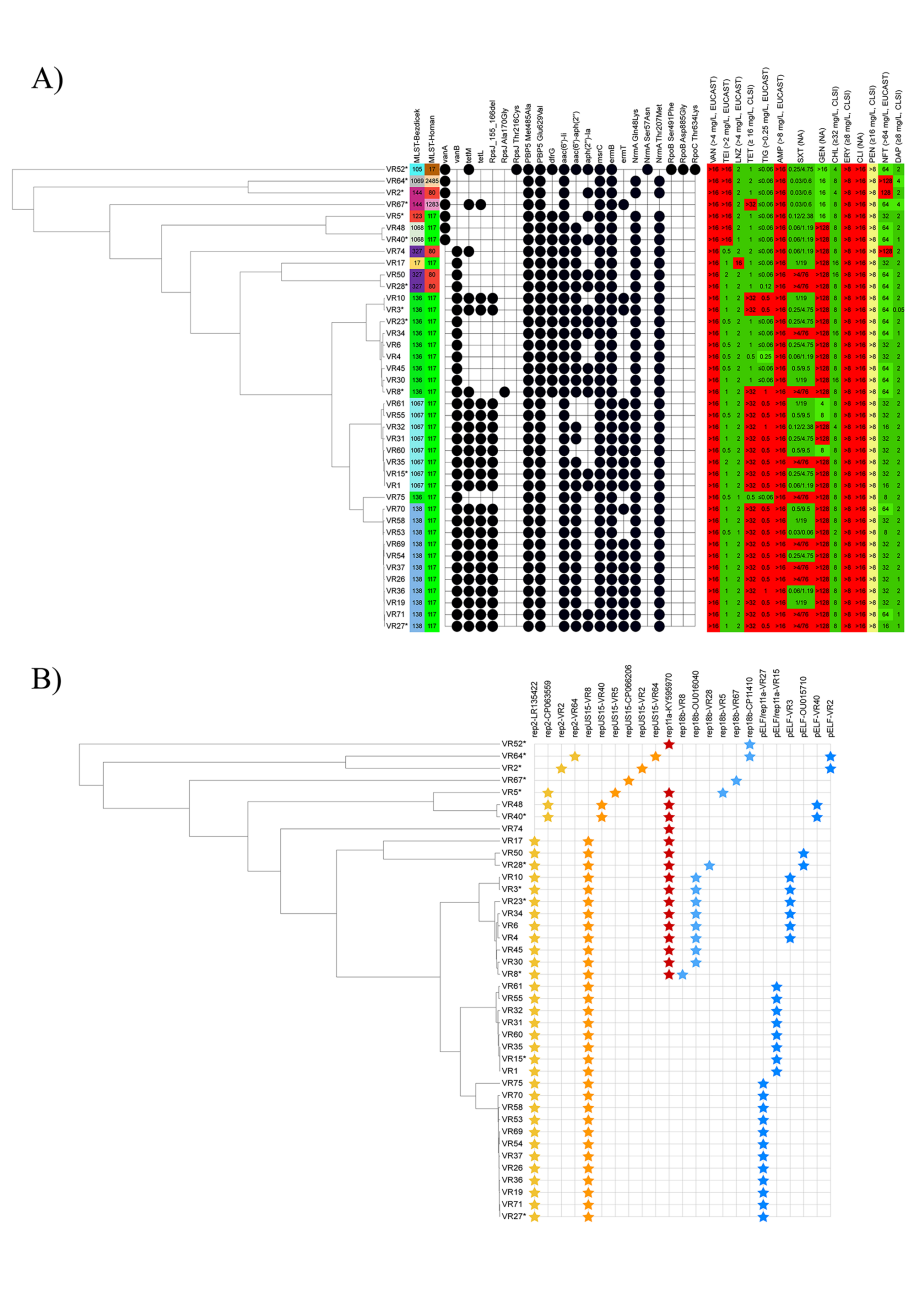
(**a**) Unrooted phylogenetic tree created using wgMLST scheme from Bionumerics v8.1 comparing 3332 loci. MLST schemes from Bezdicek et al. and Homan et al. [21, 22] and the type of *van* operon are shown for each strain. Circles denote detected acquired resistance genes and the presence of point mutations conferring resistance. A heatmap of MIC values is shown, and the colour scale is normalised for each respective breakpoint. The MIC values for penicillin are yellow, the breakpoint for penicillin is 16 mg/L (CLSI), however the testing MIC range only reached 8 mg/L. (**b**) Unrooted phylogenetic tree created using the wgMLST scheme from Bionumerics v8.1 comparing 3332 loci shows the presence of the most frequent plasmids. The rest of the plasmids are listed in the Supplementary Table 2. Colours indicate different replication genes. The stars indicate the presence of a plasmid in isolates. Isolate VR52 has an incomplete plasmid *rep*US15, isolates VR50 and VR74 had plasmids with *rep*18b and *rep*2 genes, which were detected, respectively, but their structural variants were not detected in isolates with hybrid assembly.

Among the VREfm isolates subjected to WGS, the MLST scheme from Homan et al. [21] identified 5 STs with ST117 as the predominant (*n* = 32, 80%) (Fig. 1a). In contrast, using the MLST scheme by Bezdicek et al. [22], 10 STs were found with the most frequent STs being ST138 (*n* = 11, 27.5%), ST136 (*n* = 10, 25%), ST1067 (*n* = 8, 20%) showing higher discriminatory power compared to Homan et al. scheme [21], (Figs. 1 and 2a).

wgMLST revealed a total of six clusters (Fig. 1a). Four of them comprising 2, 7, 8 and 11 isolates of the same ST (117) using the MLST scheme from Homan et al. [21]. and three different STs (136, 138 and 1067) using the MLST scheme from Bezdicek et al. [22]. (Fig. 1b). Isolates from ST138 and ST1067 clustered together; isolates from ST136 belonged to two clusters (2 and 7 isolates), and one isolate was distantly related (75 allele differences).

Considering the presence of the plasmids in four of the six clusters, identical plasmids were detected in all isolates (Fig. 2b). In the remaining two clusters, the presence of different plasmids and/or length of the plasmids with the *rep*18b gene were observed (Fig. 2b).

A list of plasmids detected in isolates with hybrid assembly is provided in the Supplementary Table 2.

### Global comparative genetic analysis of global ST117 isolates

**Fig. 3:**
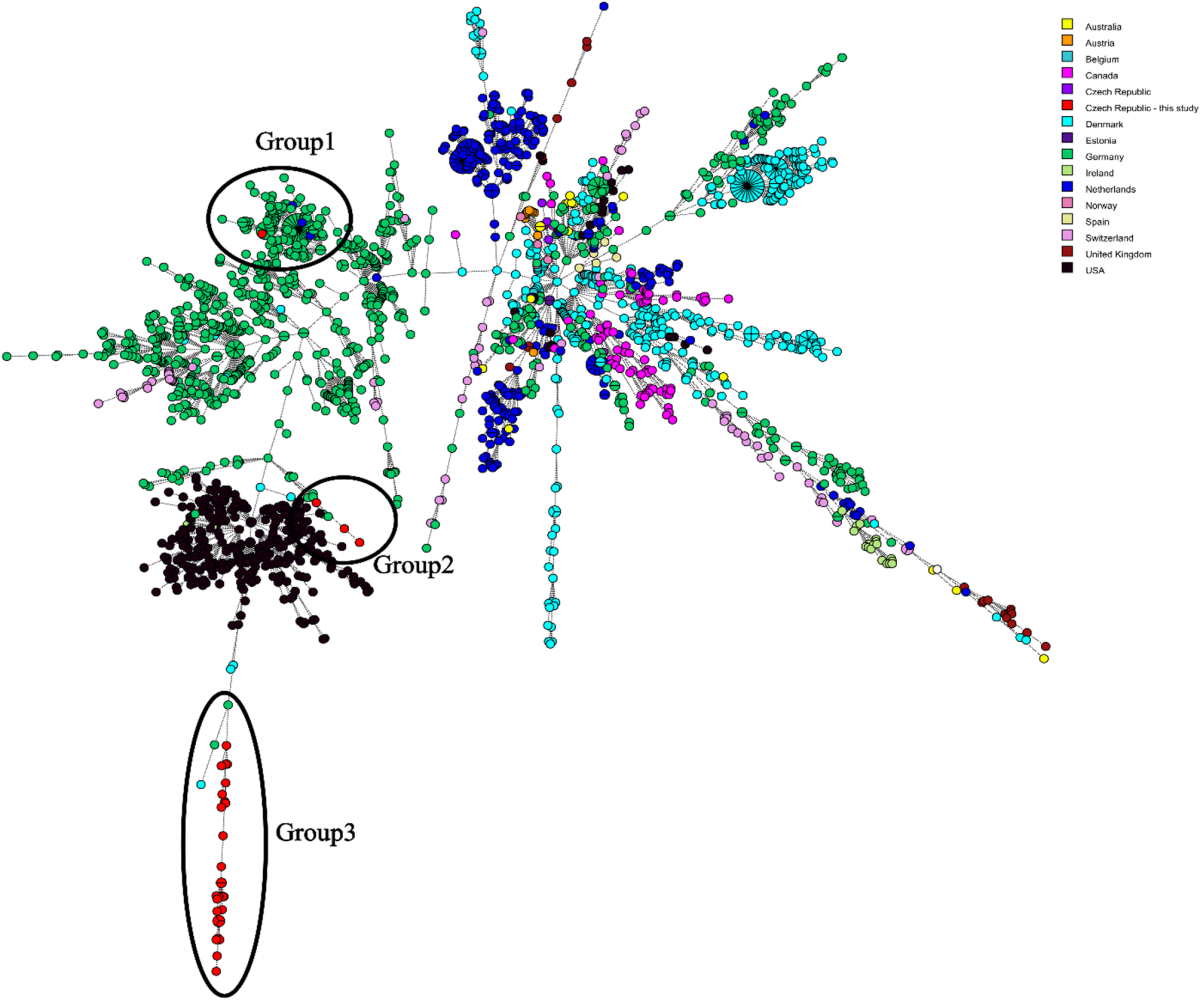
A minimum spanning tree (Bionumerics v8.1, comparing 4135 loci) comparing ST117 isolates from this study (*n* = 32) to ST117 global isolates (*n* = 2,204). Colours indicate different countries. One isolate from this study clustered closely with isolates from Germany and the Netherlands (Group 1). Groups 2 and 3 exhibit distant relatedness between isolates from this study and those from Germany and Denmark.

Only one Czech isolate from our study grouped closely with isolates from Germany and the Netherlands, differing by only 16 alleles (Group 1, Fig. 3) as determined by the wgMLST. The remaining isolates from our study were genetically distinct, differing by a minimum of 95 and 225 alleles from Groups 2 and 3, respectively (Fig. 3). When re-analysed STs using the more recently developed MLST scheme by Bezdicek et al. [22], the global ST117 isolates were subdivided into 57 distinct sequence types (Supplementary Table 3), further demonstrating the higher discriminatory power of this updated scheme.

### Antimicrobial susceptibility testing and resistance mechanisms

All 102 VREfm isolates were resistant to ampicillin and norfloxacin, with 24.51% (*n* = 25) of isolates also resistant to teicoplanin.

Among the 40 sequenced VREfm isolates, all were resistant to ampicillin (geometric mean MIC > 16 mg/L; harbouring PBP5 Met485Ala and Glu629Val substitutions), erythromycin (geometric mean MICs > 8 mg/L, harbouring *ermB* [*n* = 40], *ermT* [*n* = 20; 50%], and *msrC* [*n* = 40] genes), clindamycin (geometric mean MICs > 16 mg/L, harbouring *ermB* [*n* = 40], *ermT* [*n* = 20; 50%] genes) and norfloxacin (harbouring ParC_Ser80Ile and GyrA_Ser83Ile substitutions). Resistance to vancomycin (geometric mean MIC > 16 mg/L) was attributed to the presence of the *vanA* operon in 7 isolates (17.5%), and by the *vanB* operon in 33 isolates (82.5%). The isolates with the *vanA* operon were also resistant to teicoplanin.

Linezolid resistance was observed in one isolate, which carried a G2576T mutation in the 23S rDNA gene. The linezolid resistance was confirmed by broth microdilution (MIC = 16 mg/L). High-level gentamicin resistance (HLGR) was detected in 32 isolates (80%) which carried aminoglycoside resistance genes *aac(6’)-Ii* (*n* = 40), *aac(6’)-aph(2’’)* (*n* = 33, 82.5%), *aph(2’’)-Ia* (*n* = 16, 40%) detected either individually or in combination.

Tetracycline resistance was found in 23 isolates (57.5%) with corresponding resistance genes *tet*(M) (*n* = 23) and *tet*(L) (*n* = 22) identified either individually or in combination. Two additional tetracycline-sensitive isolates had a nonsense mutation in *tet*(M) genes, leading to premature stop codons at amino acid positions 268 or 271. Among the tetracycline-resistant isolates, 22 were also resistant to tigecycline (MIC 0.5–1 mg/L, Fig. 2a), carrying both *tet*(M) and *tet*(L) genes as well as a nucleotide deletion within the *rpsJ* ribosomal structure protein gene at nucleotide positions 155–166. One tigecycline-resistant isolate (MIC = 1 mg/L) carried the *tet*(M) gene and had a heterozygote signal in raw reads for the substitution Lys57Arg in RpsJ.

Nitrofurantoin resistance was observed in three isolates (geometric mean MIC of 40.79 mg/L, range: 8->128 mg/L). Investigation of resistance mechanisms revealed that 38 isolates (95%) carried the NmrA Gln48Lys and Thr207Met substitutions, while none harboured the EF0404 and EF0648 genes. Although the NmrA_Gln48Lys substitution and the absence of EF0404 and EF0648 genes have been linked to nitrofurantoin resistance in *E. faecium* [35, 36], isolates in this study exhibited a wide range of MICs (8->128 mg/L), including both susceptible and resistant phenotypes.

All isolates were susceptible to chloramphenicol (geometric mean of 7.86 mg/L, range 2–16 mg/L) and daptomycin (geometric mean MIC of 1.83 mg/L, range 0.5-4 mg/L). Interestingly, one daptomycin-sensitive isolate (MIC = 2 mg/L) carried the RpoB_Ser491Phe mutation that was previously associated with daptomycin resistance [15].

For trimethoprim/sulfamethoxazole, no clinical breakpoints are available; however, 11 out of 40 isolates exhibited elevated MICs (> 4/76 mg/L). The *dfrG* gene, associated with trimethoprim resistance, was detected in 17 isolates (42.5%), but the presence of the *dfrG* gene did not correlate with the observed increased MIC.

A summary of the results of antimicrobial susceptibility testing and the presence of molecular mechanisms of antimicrobial resistance is provided in Fig. 2a and the Supplementary Table 1.

### The localisation of antimicrobial resistance genes

**Fig. 4:**
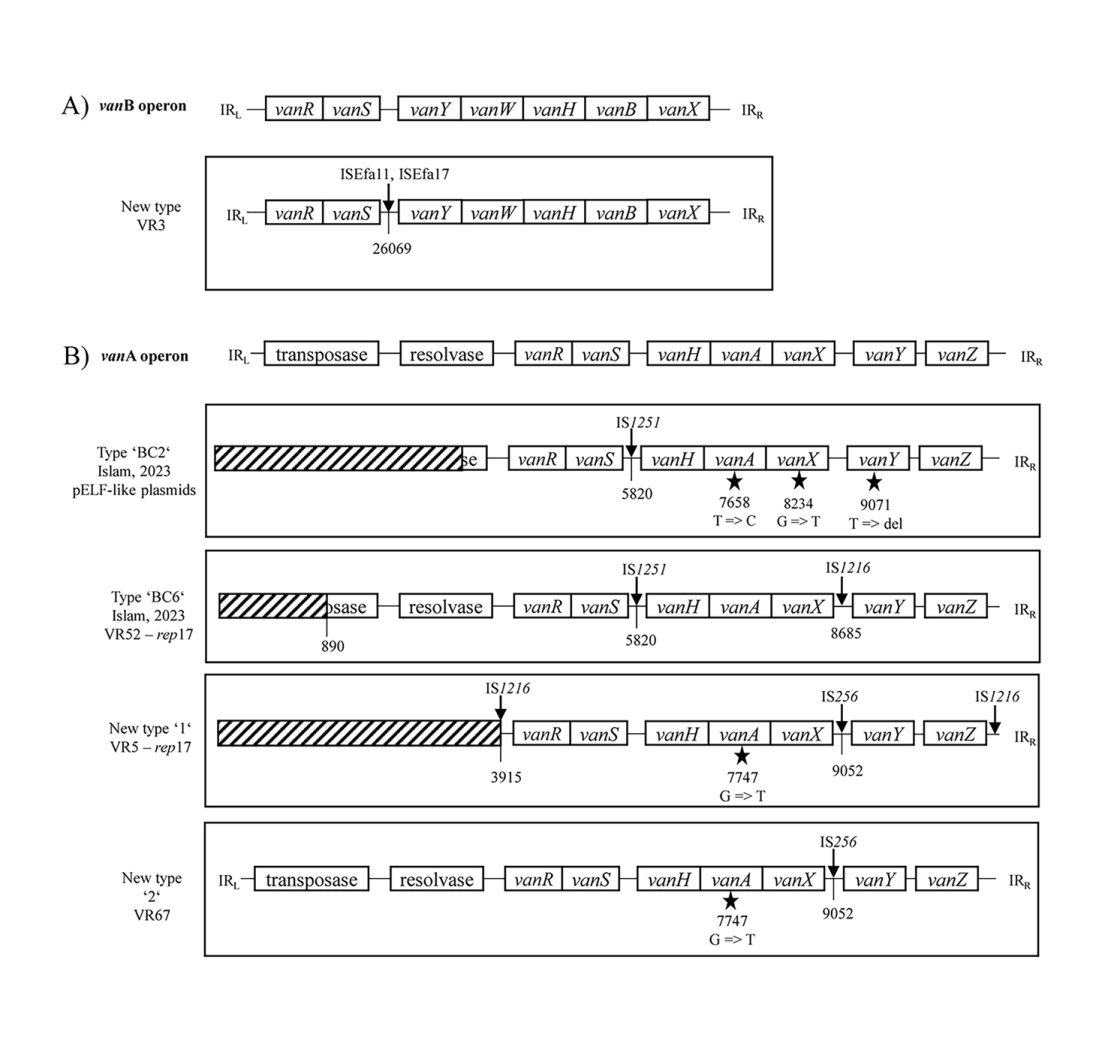
(**a**) Structural variant of Tn*1549* detected in this study. A reference of Tn*1549* (GenBank AF192329) [33] was used for comparison. (**b**) Structural variations of Tn*1546* detected in this study. A reference of Tn*1546* (GenBank M97297.1) [32] was used for comparison. Type ‘BC2’ and ‘BC6’ are from the study from Islam et al. [8]. New structural variations are deposited in GenBank under PV113235 and PV113239

The vancomycin resistance was associated with the presence of the *vanB* operon in 33 isolates (82.5%) located on the chromosome and carried a Tn*1549* transposon structural variant within two insertion sequences, ISEfa11 and ISEfa17, located between the *vanS* and *vanY* genes (Fig. 4a, GenBank accession PV113232).

In the remaining 7 isolates, the vancomycin resistance was associated with the presence of the *vanA* operon. Four isolates carried the *vanA* operon on a linear plasmid of 108.8 kb (VR2 (GenBank accession PV113235) and VR64) and 114.2 kb length (VR40 (GenBank accession PV113239) and VR48). These plasmids revealed 98.53% identity, 84% coverage (VR2 and VR64) and 97.46% identity and 89% coverage (VR40 and VR48) with a linear plasmid pELF2 (GenBank accession AP022343, Supplementary Fig. 1) [9], had a comparable GC content to pELF2 (range 33.8.-34% vs. 33.9%, respectively) and contained the *ftsK* and *repB* genes [9]. Surprisingly, these plasmids were not detected using the PlasmidFinder database [20]. Four isolates with pELF-2-like plasmid carried Tn*1546* type ‘BC2’ previously described in VREfm isolates from the USA [8]. Isolate VR52 with *rep*17 plasmid carried Tn*1546* type ‘BC6’ with different places of IS1216 insertion from the same study. In our study, two new structural variations of Tn*1546* were identified and depicted in Fig. 4b (GenBank accession PV113233 and PV113234). The remaining three isolates carried the *vanA* operon on pRUM-like plasmids, harbouring either the *rep*17 or the *rep*18b genes alone or in combination (Supplementary Fig. 2, GenBank accession PV113242, PV113248 and PV113252). However, they differed in length (24.3 kb, 41.4 kb and 58.8 kb) and GC content (35.8%, 35.1% and 35.7%, respectively).

In this set of isolates, the *aac(6’)-Ii*, *dfrG*, *ermT*, *msrC*, and *tet*(L) genes have been found solely located on the chromosome. The *tet*(M) gene was found mainly on the chromosome, with the exception of isolate VR8, which carried the *tet*(M) gene on a plasmid with the *rep*18b gene (GenBank accession PV113244). Plasmids with the *rep*2 gene dominantly carried the *ermB*, *aph(3’)*-*III* and *ant(6)-Ia* genes. Isolate VR67 carried these putative resistance determinants on a pRUM-like plasmid containing the *rep*18b gene as well as the *vanA* operon (Supplementary Table 2, GenBank accession PV113252).

## Discussion

This study aimed to address the knowledge gap on VRE epidemiological data in the Czech Republic. Although several studies have focused on VRE epidemiology in the country, only one study used WGS, but primarily for the development of a more accurate MLST scheme for *E. faecium* [22, 37]. In our study, WGS was used for detailed characterisation of 40 VREfm isolates from a tertiary care hospital.

Traditionally, MLST has been the gold standard typing method for epidemiological investigations due to its reproducibility across laboratories [38]. Moreover, sequence types can be extracted from WGS data [39]. For *E. faecium*, there are currently two MLST schemes available. The MLST scheme established by Homan *et* al. [21] was based on putative gene functions *E. faecalis*. Due to its design, this scheme often clusters distantly related isolates together [6, 24, 40]. A newer MLST scheme [22] was developed using whole genome sequencing and the most discriminative genes according to MATLAB version 2020a found in 1,649 publicly available *E. faecium* sequences after quality check control. The higher discriminative power of the new scheme was demonstrated in Japan, where it successfully differentiated two sequential outbreaks [41]. In our study, the newer scheme [22] identified 10 different STs instead of the 5 STs identified by the older scheme [21]. As a result, three clusters from wgMLST analysis were identified as ST117 by the scheme of Homan et al. [21], but isolates in these clusters belong to three different STs (ST136, ST138 and ST1067) according to the Bezdicek et al. scheme [22]. Thus, we recommend adopting this new scheme due to its higher discriminatory power.

In our study, we identified three predominant STs (136, 138 and 1067), contrasting to Czech data from Bezdicek et al., who reported ST42 and ST140 as predominant [22]. These differences may reflect the geographical location of the studied hospitals (over 200 km) and suggest the need for a multicentre study to map the VRE epidemiology in Czech hospitals.

To compare our epidemiological data with other studies, we refer to STs identified using the Holman et al. scheme, as the new MLST scheme was published in 2023. Similar to our findings, ST80 and ST117 were identified as dominant in Europe [42, 43, 44], and in the Czech Republic [22, 37]. Over time, these STs have gradually replaced others, such as ST203, which remains dominant in Ireland [42].

When isolates from the predominant STs in this study were compared with global ST117 isolates, only one isolate from this study clustered with isolates from Germany and the Netherlands (Fig. 3), suggesting the presence of a distinct local Czech clone. This is further supported by the non-relatedness of isolates from this study to other Czech isolates downloaded from the Pathogenwatch database, which originated from a different hospital. However, additional isolates from other Czech hospitals are needed to confirm these findings and determine the extent of local clonal dissemination.

In Europe, vancomycin resistance in *E. faecium* was historically dominated by the *vanA* operon but has now shifted to *vanB* [42, 43, 44]. In the Czech Republic, the *vanA* operon was reported as the predominant vancomycin resistance mechanism, however, the investigated isolates were collected between 2005 and 2019 [37, 45]. In our study, the *vanB* operon was the most prevalent mechanism of vancomycin resistance, though this finding may be biased, as only 39.2% of isolates were selected for WGS. Nevertheless, susceptibility testing data support the *vanB* operon predominance, as 71% of non-sequenced isolates were sensitive to teicoplanin. However, a multicentre study is needed to determine the predominant mechanism of vancomycin resistance in the Czech Republic.

Short-read sequencing is commonly used for surveillance but has limitations, particularly in detecting structural variants [46]. These limitations can be addressed with long-read sequencing, which offers significant advantages, such as the ability to identify plasmids and other mobile genetic elements [47]. While long-read sequencing is frequently used for Gram-negative bacterial species [46, 47], its application to Gram-positive bacteria remains uncommon, resulting in limited data availability. This, in turn, restricts bioinformatic tools and databases for analysing submitted WGS data. In our study, we used hybrid assemblies combined with a Blast search to identify putative plasmids, revealing several plasmids and their variants, whose sequences have now been deposited in the GenBank. In addition to plasmids, we also analysed transposons associated with *van* operon carriage.

Databases like the Transposon Registry available at https://transposon.lstmed.ac.uk/ [48], provide valuable resources; however, transposons carrying *vanA* Tn*1546* and *vanB* Tn*1549* operons lack unified nomenclature and structural variation records, complicating differentiation between new and known variants. In this study, the *vanB* operon was localised on the chromosome within a Tn*1549* structural variant featuring two insertion sequences, ISEfa11 and ISEfa17, inserted between the *vanS* and *vanY* genes (Fig. 4b, GenBank accession PV113232). The *vanA* operon was localised on plasmids, both linear and circular, consistent with prior findings [8, 9]. Two previously described structural variants of Tn*1546* were detected, including the BC2 type [8] on pELF-like plasmids. Interestingly, this variant has not been identified on pELF plasmids in studies from Japan and Ireland [8, 42]. Additionally, two novel Tn*1546* structural variations were described in our study (GenBank accession PV113233 and PV113234). One of the novel Tn*1546* structural variations was carried on a plasmid containing two different *rep* genes, *rep*17 and *rep*18b (GenBank accession PV113242). This type of plasmid was also found in a study by Islam et al. [8] in Texas, USA.

Currently, the treatment options for VRE infections are limited to linezolid, tigecycline or daptomycin. Despite the increasing use of linezolid and the rise in linezolid-resistant isolates reported in recent years [49, 50], we detected only one resistant isolate in our study. Linezolid resistance in this isolate was encoded by the G2576U mutation in the 23 rRNA, the most common resistance mechanism associated with linezolid resistance [10]. However, antimicrobial susceptibility testing of *E. faecium* isolates collected between 2009 and 2019 at the Czech National Reference Laboratory for Antibiotics revealed that 13.4% of isolates were resistant to linezolid [37].

In contrast, the occurrence of tigecycline resistance in VRE has been reported only sporadically [11, 37]. However, in our study, tigecycline resistance was observed in 21.6% of isolates (22/102), a high prevalence that is likely associated with the spread of closely related strains within the hospital.

Resistance mechanisms included the presence of *tet*(M) and *tet*(L) genes, along with a nucleotide deletion in the *rpsJ* gene at positions 155–166, which have been previously linked to tigecycline resistance [13, 19]. One tigecycline-resistant isolate carried only the *tet*(M) gene and exhibited a heterozygous signal in the raw reads for the substitution Lys57Arg in RpsJ. This isolate differed from others in that the *tet*(M) gene was located on a plasmid containing the *rep*18b gene (GenBank accession PV113244), whereas in the remaining isolates, the *tet*(M) gene was found on the chromosome.

By combining WGS and antimicrobial susceptibility testing in our study, we observed several discrepancies between resistance genotypes and phenotypes. Even though all the 40 isolates were susceptible to daptomycin, one isolate carried the RpoB_Ser491Phe substitution, which has been previously associated with daptomycin resistance [15]. Further, the *dfrG* gene associated with trimethoprim resistance was present in 17 isolates, but elevated MICs to trimethoprim-sulfamethoxazole (> 4/76 mg/L, 11 isolates) did not correlate with susceptibility phenotype. This discrepancy may be due to the fact that we tested trimethoprim-sulfamethoxazole as a combination rather than trimethoprim alone. When trimethoprim is combined with sulfamethoxazole, their synergistic effect can enhance antibacterial activity, potentially masking resistance that might be observed when each drug is tested individually [51, 52].

Interestingly, three isolates were resistant to nitrofurantoin. However, when analysing resistance mechanisms, we found that most isolates, including susceptible ones, carried the NmrA_Gln48Lys substitution and lacked the EF0404 and EF0648 genes, which have been previously identified as resistance mechanisms in *E. faecium* [35, 36]. This suggests that other, yet unidentified, mechanisms may be contributing to the observed resistance.

## Conclusion

The spread of tigecycline and vancomycin-resistant *E. faecium* ST117 with the *vanB* operon as the predominant mechanism of vancomycin resistance was identified. The discrepancies between resistance genotypes and phenotypes highlight the importance of integrating genomic and phenotypic surveillance in antimicrobial resistance monitoring. Ongoing monitoring of plasmid- and transposon-mediated resistance elements is essential to understand the evolving epidemiology of these highly resistant enterococcal strains.

## Electronic supplementary material

Below is the link to the electronic supplementary material.

Supplementary Material 1

Supplementary Material 2

## Data Availability

Raw sequence data and hybrid assemblies generated in this study are publicly available under the BioProject PRJNA1220344 (release date after publication).

## Abbreviations

CGE: Center for Genomic Epidemiology
CLSI: Clinical and Laboratory Standards Institute
DDM: Disk diffusion method
EARS-net: European Antimicrobial Resistance Surveillance Network
EUCAST: European Committee on Antimicrobial Susceptibility Testing
HLGR: High-level gentamicin resistance
MIC: Minimum inhibitory concentration
MLST: Multilocus sequence typing
ST: Sequence type
VRE: Vancomycin-resistant enterococci
VREfm: Vancomycin-resistant* Enterococcus faecium*
wgMLST: Whole genome multilocus sequence typing
WGS: Whole genome sequencing
WHO: World Health Organization
