# Supplementary material for: Genomic insights into the spread of vancomycin- and tigecycline-resistant Enterococcus faecium ST117
Source: Ann Clin Microbiol Antimicrob. 2025 Jun 11;24:36. doi: 10.1186/s12941-025-00806-7 (PMC12153105; doi:10.1186/s12941-025-00806-7)
Supplement: Supplementary file 1 — Supplementary Material 1 [file 12941_2025_806_MOESM1_ESM.docx]

**Supplementary Figures**


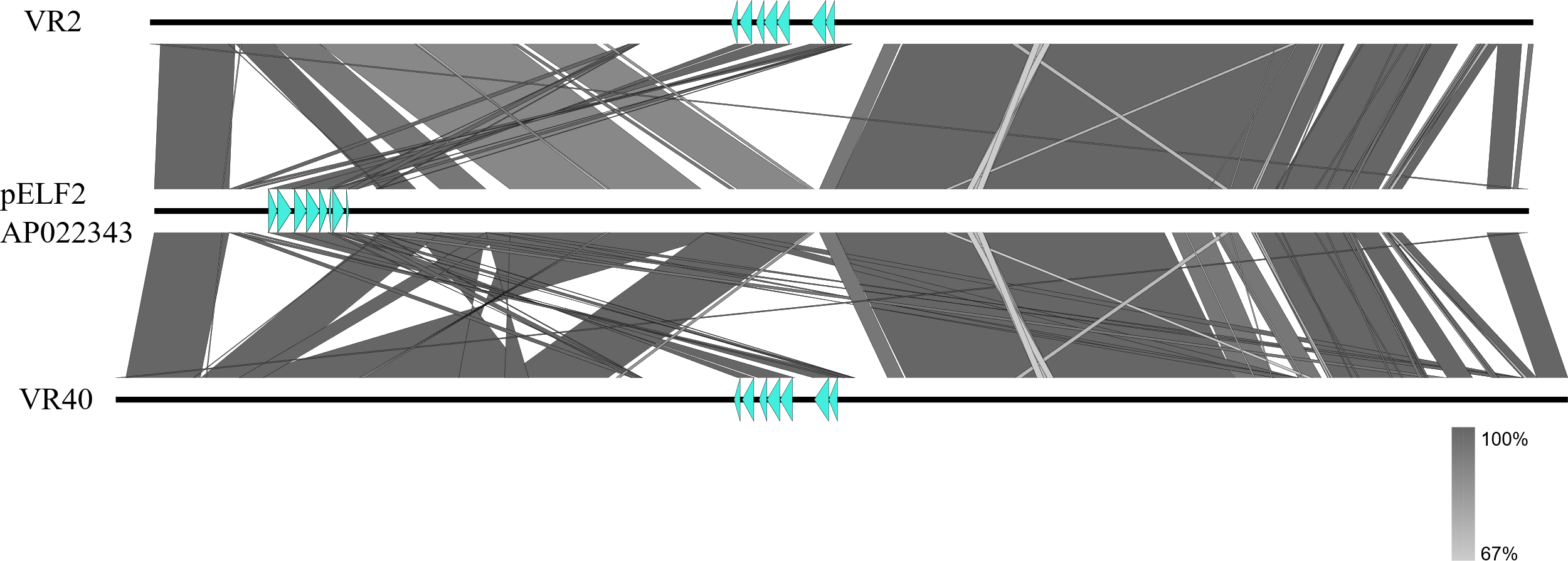


Supplementary Figure 1. Comparison of plasmid sequences of VREfm isolates VR2-pELF2-VR40 with *van*A operon highlighted in blue. pELF2-like plasmids of VR2 and VR64 are identical. A reference of plasmid pELF2 (NCBI accession AP022343) [9] was used for comparison.


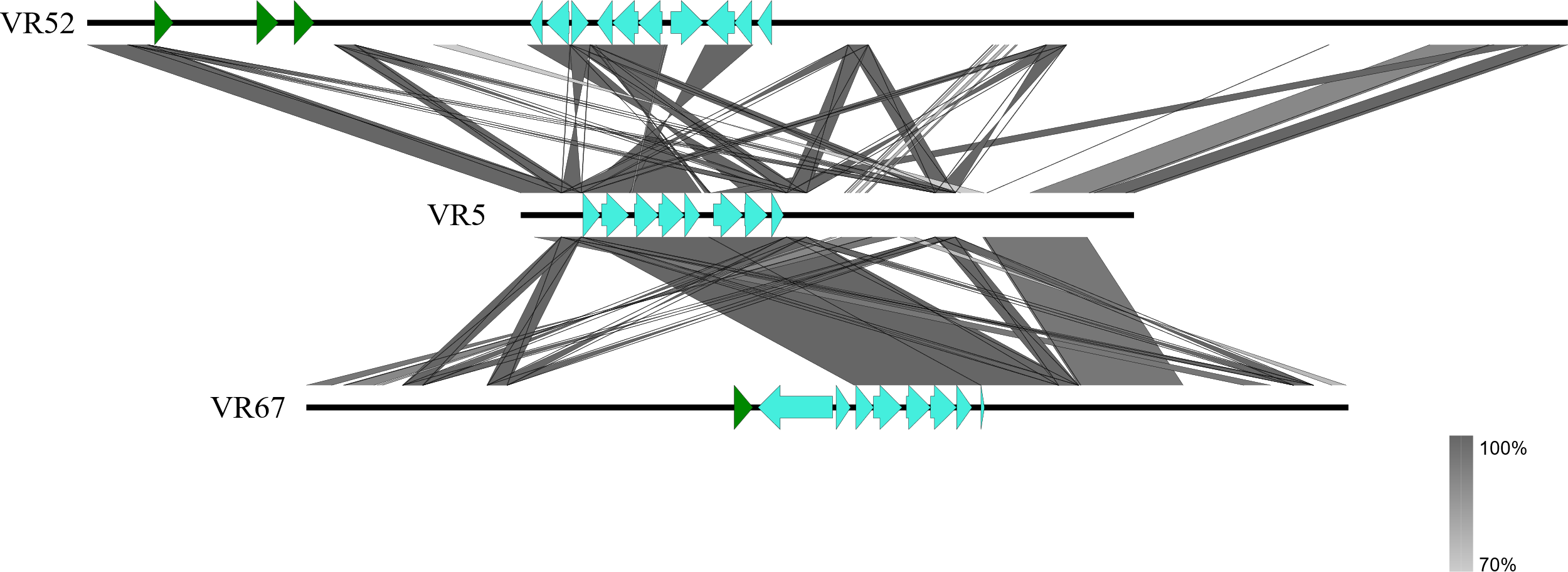


Supplementary Figure 2. Comparison of plasmid sequences of VREfm isolates VR52-VR5-VR67 carrying pRUM-like plasmids with Tn*1546* (including *van*A operon) highlighted in blue. Isolate VR5 harboured Tn*1546* on “mosaic” plasmid: harboured both *rep*17 and *rep*18b genes. Isolate VR52 harboured Tn*1546* on a plasmid with the *rep*17 gene. The same plasmid also harboured *ant(6)-Ia, aph(3')-III* and *erm*B gene, highlighted in green. Isolate VR67 harboured the *rep*18b gene and *erm*B gene*,* which is highlighted in green.
